# The effect of carbohydrate mouth rinse on performance, biochemical and psychophysiological variables during a cycling time trial: a crossover randomized trial

**DOI:** 10.1186/s12970-018-0225-z

**Published:** 2018-05-02

**Authors:** Amanda M. J. Ferreira, Luiz F. Farias-Junior, Thaynan A. A. Mota, Hassan M. Elsangedy, Aline Marcadenti, Telma M. A. M. Lemos, Alexandre H. Okano, Ana P. T. Fayh

**Affiliations:** 1 Graduate Progrtam in Physical Education, Federal University of Rio Grande do Norte, Avenida senador Salgado Filho 3000, Campus Universitário, Lagoa Nova, Natal, RN Brazil; 2 HCor, Institute of Research, Coracao Hospital, São Paulo, SP Brazil; 3 IC/FUC, Postgraduate Program in Health Sciences, Cardiology Institute / University Foundation of Rio Grande do Sul Cardiology, Porto Alegre, RS Brazil; 4 UFABC, Universidade Federal do ABC, São Bernardo do Campo, São Paulo, Brazil

**Keywords:** Carbohydrate mouth rinse, Exercise, Physical performance, Perceived exertion, Cycling

## Abstract

**Background:**

The hypothesis of the central effect of carbohydrate mouth rinse (CMR) on performance improvement in a fed state has not been established, and its psychophysiological responses have not yet been described. The aim of this study was to evaluate the effect of CMR in athletes fed state on performance, biochemical and psychophysiological responses compared to ad libitum water intake.

**Methods:**

Eleven trained male cyclists completed a randomized, crossover trial, which consisted of a 30 km cycle ergometer at self-selected intensity and in a fed state. Subjects were under random influence of the following interventions: CMR with a 6% unflavored maltodextrin solution; mouth rinsing with a placebo solution (PMR); drinking “ad libitum” (DAL). The time for completion of the test (min), heart rate (bpm) and power (watts), rating of perceived exertion (RPE), affective response, blood glucose (mg/dL) and lactate (mmol/DL), were evaluated before, during and immediately after the test, while insulin (uIL/mL), cortisol (μg/dL) and creatine kinase (U/L) levels were measured before, immediately after the test and 30 min after the test.

**Results:**

Time for completion of the 30 km trial did not differ significantly among CMR, PMR and DAL interventions (means = 54.5 ± 2.9, 54.7 ± 2.9 and 54.5 ± 2.5 min, respectively; *p* = 0.82). RPE and affective response were higher in DAL intervention (*p* < 0.01). Glucose, insulin, cortisol and creatine kinase responses showed no significant difference among interventions.

**Conclusions:**

In a fed state, CMR has not caused metabolic changes, and it has not improved physical performance compared to ad libitum water intake, but demonstrated a possible central effect. ReBec registration number: RBR-4vpwkg. Available in http://www.ensaiosclinicos.gov.br/rg/?q=RBR-4vpwkg.

## Background

Nowadays, sport and exercise science have advanced in the knowledge about ergogenic use of carbohydrates for delay or reduction of central fatigue [1, 2]. To date, the ergogenic effects of carbohydrate intake and its physiological responses, including hormones secretion are well-established during prolonged exercise [3, 4]. However, in short duration and high intensity cycling exercises or in first hour of exercise [5, 6] the carbohydrate intake is not recommend because to high muscle glycogen availability [7]. Thus, main strategy adopted to improve the performance had been the preparation before competitions and use of hydration strategy [8]. The drinking ad libitum (DAL) strategy, in which there is a free-choice fluid intake, has been well-suited to high-intensity cycling exercises for maintaining hydration status [9–11], particularly in time trials [10].

From another perspective, a hypothesis has emerged that rinsing the mouth with a solution of carbohydrates without swallowing has an ergogenic effect in these modalities. The study by Carter et al. [12] was the pioneer and observed that there was an improvement in performance when cyclists adopted CMR without swallowing during a 1 h time trial at relatively high intensity (75% W_max_). Afterward, other studies too reported an enhanced performance after CMR on 60 min trial [13–17]. In this sense, the CMR strategy was included in the last official positioning of the American College of Sports Medicine for improvement of physical performance in exercise with duration less than 1 h [1]. The possible mechanism responsible by improved performance after CMR is detection of carbohydrate by taste receptors in the oral cavity send afferent neural signals directly to the brain, activating brain regions presumed to be involved in reward and motor control, regardless of their absorption by the gastrointestinal tract [2, 14, 18, 19]. However, in most of these studies that found effect positive, the athletes were exposed to the CMR being fasted [12, 13, 17, 20], and it is not common among athletes before exercise. Contrarily, other studies showed no effect of CMR on performance in a fed state compared to mouth rising with placebo solution or solution with different carbohydrate concentration [21–23].

These findings suggest that effect of CMR on performance improvement in a fed state remains unclear. To date, is not know the effect of CMR on hormones secretion related to carbohydrate consumption (eg. cortisol and insulin), as well, the effect of CMR with athletes in a fed state on perceived exertion and affective response during time trail test also not described. Additionally, there is still no study comparing the CMR strategy with traditional recommendations for cycling in this type of test, in which individuals are fed and are guided about hydration strategies [10]. Therefore, the aim of the present study was to evaluate biochemical and psychophysiological responses under the CMR strategy in individuals at a fed state, compared to DAL and a placebo.

## Methods

### Recruitment and participants

Fourteen male trained cyclists were recruited to participate in the study. Participants were cycling for at least 150 km about 5 h/wk. and were heat acclimated. Exclusion criteria included smoking, previous diagnosis of chronic diseases and musculoskeletal injuries within the last 6 months that could interfere with training routine. The recruitment occurred through the dissemination of the study in digital media and personal contact, in the Department of Physical Education of the Federal University of Rio Grande do Norte and in cycling teams of the city of Natal-RN, Brazil, that meet periodically in several places of the city. This study was approved by the Research Ethics Committee of the Federal University of Rio Grande do Norte under protocol number 31747714.7.0000.5568. All participants were informed about the procedures and signed an informed consent form prior to enrollment in the study. The final number of participants included in the present study was based on the study of Carter et al. [12]. Sample size was calculated with a test power of 90% and significance level of 5%. The protocol of this randomized crossover clinical trial was registered on a publicly accessible database (http://www.ensaiosclinicos.gov.br/rg/RBR-4vpwkg/).

### Study design

Each subject attended the laboratory on five different occasions. The first visit aimed to characterize subjects by application of an anamnesis form, collection of anthropometric data and evaluation of performance in an incremental exercise test to exhaustion. The second visit was a familiarization session with the experimental procedures. In the 3th to 5th visits, in random and cross order, the participants completed a 30 km cycle ergometer time trial. All tests were performed in the afternoon and in an acclimatized room, with control of temperature and humidity (average temperature of 22.1 °C and humidity of 72.5%). Participants were instructed to abstain from alcohol, caffeine, tobacco and exercise, and also to stay properly hydrated for 24 h prior each visit. It was mandatory the ingestion of 500 mL of fluid 2 h before the tests to ensure that participants were hydrated, according to the recommendations from the American College of Sports Medicine [24]. All test sessions were conducted at the same time of the day and subjects were in a fed state with a minimum of 2–3 h from the last meal. Participants were also instructed not to change your eating pattern during the study and in the days leading up to the tests, particularly the last meal prior testing. In addition, they were advised to record all foods and beverages consumed throughout the day, including type and amount of food, and time of consumption in a diary. Based on this food diary, nutritional composition was analyzed using the software (DietWin®, 2015).

### Anthropometric evaluation

We evaluated the following anthropometric parameters: body mass, height, and skinfolds. Body mass (kg) and height (m) was determined by a portable digital scale coupled to a stadiometer Welmy® (W 110 H, Santa Bárbara d’Oeste, SP, Brasil), with accuracy of 0.1 kg and 0.01 cm, respectively. These parameters were used to calculate the body mass index (BMI, kg/m^2^) for classification of the nutritional status according to the cut-off points defined by the World Health Organization [25]. All measurements of skinfolds (triceps, subscapular, chest, biceps, iliac crest, abdominal, medial thigh and calf) were made on the right side of the body by using a compass (Cescorf®) with accuracy of 0.1 mm. Skinfold measurements were used for calculation of body density using the generalized equation proposed by Jackson and Pollock [26] and later converted to fat percentage, according to formula proposed by Siri [27].

### Incremental exercise test

Participants performed an incremental exercise test on cycle ergometer (Velotron, Racermate, Inc., Seattle, WA, USA) to determine maximum heart rate (HRmax), peak oxygen uptake (VO_2_peak) and peak power output (Wmax). The protocol of test began at 100 W for 1 min followed by fixed increments of 25 W per minute until volitional exhaustion or cadence lower that 80 rpm. Heart rate (beats/minute) was continuously recorded throughout the test using a Polar Monitoring System (RS800cx, Polar Electro®, Oy, Kempele, Finland). Oxygen uptake was continuously recorded using a breath-by-breath gas exchange automatic system (Quark - CPET, Cosmed, Roma, Italy). Wmax was defined as the power output reached during the last full stage completed before volitional exhaustion. HRmax was considered the greater HR achieved during the test [28]. The higher mean oxygen uptake of 30 s during incremental exercise test was defined as the VO_2_peak [28].

### Experimental interventions

Participants were under random influence of the following interventions in a fed state: carbohydrate mouth rinse (CMR), placebo mouth rinse (PMR), and drinking “ad libitum” (DAL). The CMR intervention consisted of a solution for carbohydrate mouth rinse comprised 6.4% of unflavored maltodextrin (Atlhetica®), in accordance with recommendations from literature [6]. Subjects received 25 ml of the solution in a cup after every 3.75 km (12.5% of the time trial completed), replicating protocols used in this area of investigation [7, 23, 29]. They were instructed to rinse the fluid around their mouths for about 10 s, and then, spit the whole solution in a container indicated by the investigator. The expelled volume of liquid was measured to ensure that ingestion of solution was not significant. The PMR intervention consisted of a placebo solution prepared from artificial sweetener sucralose in powdered form, concentration of 0.08 g/*L. prior* to the beginning of protocol, we performed sensorial analysis tests to adjust the taste of the placebo solution, to ensure that the interventions had similar taste. The DAL intervention consisted of water availability as much as the subjects wanted (ad libitum) in cups of 150 mL, and the volume ingested was registered.

### 30 km time trial test

Participants performed the 30 km time trial tests using a cycle ergometer coupled to the computer (Velotron, Racermate, Inc., Seattle, WA, USA). Participants were allowed to pedal at a preferred cadence and were encouraged to finish the 30 km time trial in lower time as possible. During the 30 km time trial tests, cadence (rpm), speed (km/h), and power output (W) were recorded by software coupled to cycle ergometer. Further, heart rate (HR, bpm) was monitored using a Polar Monitoring System (Polar RS800cx, Kempele, Finlândia). Every 7.5 Km (25% of the time trial completed), the rating perceived exertion was recorded using scale of RPE proposed by Borg [30]. In the same moment, the affective response was recorded using the Feeling Scale proposed by Hardy and Rejeski [31] for monitor level of pleasure or displeasure during exercise. The time to complete the 30 km time trail tests were recorded by software coupled to cycle ergometer. Participants were not notified about their times to complete time trials in any session throughout the study period as well as they were not informed about performance variables during all time trials tests. In each time trial test, the weight loss (%) was calculated by weight after minus weight before time trail divided by weight before.

### Biochemical variables

Biochemical markers assessments were creatine kinase, insulin and cortisol. Blood samples (10 ml) were collected from an antecubital vein by a trained professional using syringe and disposable needles. Blood samples were collected immediately before, immediately after and 30 min after time trail cessation, in heparin containing tubes and centrifuged at 4000 rpm and 4 °C. Plasma aliquots were stored at − 75 °C to posterior analysis. Biochemical analyses were performed in duplicate using standard commercial kits (Labtest®, Minas Gerais, Brazil). Plasma insulin (uIL/mL) and cortisol (μg/dL) concentration were measured by chemiluminescence (Immulite® 1000 Imunoassay System, Siemens, Germany), with maximum intra-assay coefficient of variation was 4.8 and 7 for repeat samples, respectively. Creatine kinase (U/L) levels were measured by the electroenzymatic method (Labtest®, Minas Gerais, Brazil), with maximum intra-assay coefficient of variation of 6%. For reduce inter and intra-assay variance during analysis, the same laboratory technician perform all analysis. All pipettes were properly calibrated and were pre-wetting tip in the solution to be pipetted. Additionally, before and during the time trial tests every 10 km completed, the glucose (mg/dL) and lactate (mmol/dL) were monitored. For this, blood samples (0.3 μL) were taken from the fingertip and analyzed immediately for blood lactate concentration using reagent strips (Accutrend®, Roche, German).

### Statistical analysis

Data were recorded in a database created on Microsoft Excel® version 2013. Statistical analysis was performed using SPSS® 22.0 software, and the carry-over effect was evaluated using the STATA 1.2, both software for Windows. Continuous variables were described as mean ± standard deviation, and categorical variables as absolute numbers and percentages. The variables of time, average heart rate, average speed, average power output, energy consumption and food consumption, regardless of time and intervention, were analyzed by comparison of means using repeated measures ANOVA (RM-ANOVA). The variables measured in more than one moment during the trial such as weight, heart rate, power, perceived exertion, affective response, and plasma levels of biochemical markers were evaluated according to intervention and time using the Generalized Estimating Equation (GEE: General Estimation Equation). Normal probability distribution for symmetrical variables and gamma probability distribution for asymmetric variables, adjusted for the Bonferroni test. The level of statistical significance was accepted as *p* < 0.05.

## Results

In total, eleven male trained cyclists participated in this study. The characteristics of the participants are presented in Table 1. Table 2 shows the food consumption in the 3 days of the experimental procedures. No statistically significant difference was observed for energy and nutrients in 24 h and in the meal before test.

**Table 1:** Characteristics of the sample (*n* = 11)

Variables	Mean ± SD
Age (years)	30.4 ± 6.2
Body mass (kg)	73.6 ± 7.8
Height (m)	1.73 ± 0.08
Body Mass Index (kg/m^2^)	24.7 ± 2.2
Body Fat Percentage (%)	11.2 ± 3.4
Maximum oxygen consumption (ml/kg/min)	55.3 ± 5.6
Maximum power (Watts)	362.5 ± 48.9
Maximum heart rate (bpm)	187.5 ± 19.8

**Table 2:** Food intake of athletes in three different interventions

Food intake	CMR	PMR	DAL	*p*
24 h before the test				
Energy intake (Kcal)	2454 ± 753	2682 ± 960	2965 ± 1183	0.599
CHO intake (g)	307.4 ± 109.2	302.7 ± 90.7	359.4 ± 150.9	0.593
% de contribution	50	45	48	
g/kg	4.2	4.1	4.9	
PTN intake (g)	144.0 ± 44.8	171.8 ± 128.2	196.6 ± 128.4	0.307
% de contribution	23.5	23.5	26.5	
g/kg	2.0	2.3	2.7	
LIP intake (g)	72.0 ± 38.1	87.1 ± 20.5	82.3 ± 33.6	0.565
% de contribution	26	30	25	
g/kg	1.0	1.2	1.1	
Meal pre to the test				
Energy intake (Kcal)	669 ± 137	643 ± 83	621 ± 93	0.288
CHO intake (g)	74.6 ± 20.1	62.4 ± 12.9	68.2 ± 18.8	0.107
% de contribution	45	39	44	
g/kg	1.0	0.8	0.9	
PTN intake (g)	62.9 ± 21.4	53.8 ± 8.5	57.2 ± 16.1	0.462
% de contribution	38	34	37	
g/kg	0.9	0.7	0.8	
LIP intake (g)	12.2 ± 4.1	18.6 ± 8.5	12.5 ± 5.7	0.106
% de contribution	16	25	18	
g/kg	0.2	0.3	0.2	

Data were analyzed with RM-ANOVA. Values expressed in Mean ± SD

*CMR* carbohydrate mouth rinsing, *PMR* placebo mouth rinsing, *DAL* drinking ad libitum, *CHO* carbohydrates, *PTN* proteins, *LIP* lipids

The performance of participants was similar among interventions, considering the time for completion of the trial, average power, average speed and average heart rate (*p* > 0.05). Performance results during 30 km cycling time trial are shown in Table 3. As expected, 30 time trial tests were performed at high intensity (i.e. 81.4% maximum HR).

**Table 3:** Comparison of performance in cycling 30 km time trial test among three interventions

Variables	CMR	PMR	DAL	*p*
Time to complete (min)	54.5 ± 2.9	54.7 ± 2.9	54.5 ± 2.5	0.82
Avarage power (W)	198.6 ± 25.9	196.6 ± 26.9	196.9 ± 22.4	0.84
Avarage speed (km/h)	33.1 ± 1.3	32.9 ± 1.8	32.9 ± 1.5	0.84
Avarage heart rate (bpm)	153.9 ± 13.0	150.1 ± 13.6	153.7 ± 16.5	0.38

Data were analyzed with RM-ANOVA. Values expressed in Mean ± SD

*CMR* carbohydrate mouth rinsing, *PMR* placebo mouth rinsing, *DAL* drinking ad libitum

**Fig. 1:**
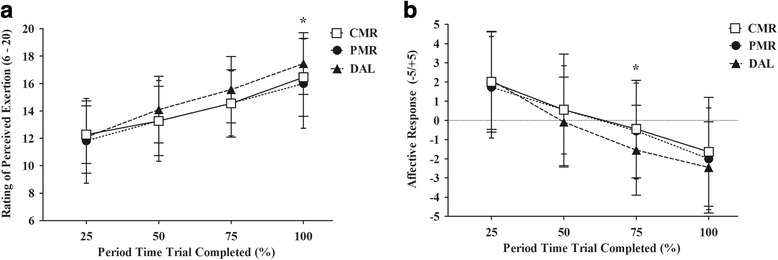
Comparison of rating perceived exertion (Panel **a**) and affect response (Panel **b**) during 30 km time trial test among three experimental treatments. CMR, carbohydrate mouth rinsing. PMR, placebo mouth rinsing. DAL, drinking ad libitum. Data expressed in mean ± SD. Analyzed data according to GEE. *Time and intervention interactions was *p* < 0.01 for both

The RPE increased significantly in all interventions throughout the trial (*p* < 0.01). In addition, there was a significant interaction between time of trial and interventions (*p* = 0.01). In the last 7.5 km of time trial in DAL intervention, the RPE was higher compared to CMR (*p* = 0.04) and PMR (*p* = 0.01) (Fig. 1a). The affective response decreased significantly during time trial in all interventions (*p* < 0.01). Furthermore, there was a significant interaction between time and interventions when considering the affective response (*p* < 0.01). In the last 15 km of the time trial, the affective response was more negative in DAL intervention compared to CMR and PMR (*p* = 0.01, *p* = 0.07, respectively) (Fig. 1b).

Fluid intake during time trial averaged at 10.8 ± 4.5 mL and 10.6 ± 8.7 mL for CMR and PMR interventions, respectively, and they were statistically lower (*p* < 0.01) compared to DAL intervention that averaged at 433.2 ± 258.8 mL. In CMR and PMR interventions, fluid intake corresponded to the volume that was not expelled after rinsing. In all interventions, cyclists presented weight loss (CMR = 1.7%; PMR = 1.9%; DAL = 1.4%), but DAL intervention showed the lowest weight loss percentage (*p* < 0.01).

**Fig. 2:**
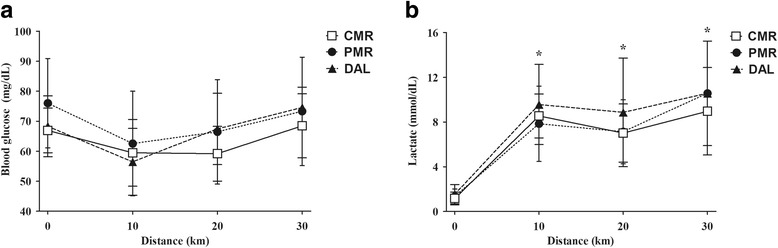
Behavior of glycemia (Panel **a**) and blood lactate (Panel **b**) during the time trial in the three treatments. CMR, carbohydrate mouth rinsing. PMR, placebo mouth rinsing. DAL, drinking ad libitum. Data expressed in mean ± SD. Analyzed data according to GEE. *Time and intervention interactions was *p* < 0.01

During the 30 km time trials, blood glucose levels showed no significant change, but its concentration decreased at first and increased in the final kilometers, without interaction between intervention and time (Fig. 2a). There was significantly time and treatment interactions for plasma lactate concentration (*p* < 0.01). Plasma lactate concentration was higher in DAL intervention compared to CMR (*p* = 0.01) and PMR (*p* = 0.08) (Fig. 2b). The peak of lactate occurred when athletes showed intense effort during the trial.

The results of biochemical response are shown in Table 4. A creatine kinase (CK) tended to increase throughout the trial, but did not change significantly over time and among interventions. Cortisol also tended to increase across the trial (*p* = 0.06), showing an interaction between intervention and time, but no significant difference among interventions. Finally, insulin concentration showed no interaction between time and intervention during time trial (*p* = 0.66), but the lowest levels occurred at 60 min of trial under PMR intervention.

**Table 4:** Comparison of cortisol, insulin and creatine kinase (CK) before (0’), the end (60’) and 30 min after the end (90) of three interventions in time trial

Variables	Interventions	0’	60’	90’	*p* ^a^	*p* ^b^	*p* ^c^
Cortisol (μg/dL)	CMR	11.2 ± 4.3*	17.0 ± 8.32	18.8 ± 6.5	0.056	0.566	0.047
	PMR	8.5 ± 3.7^*^	17.0 ± 6.73	17.5 ± 5.9			
	DAL	11.5 ± 7.5	18.2 ± 8.39	17.7 ± 6.3			
Insulin (uIL/mL)	CMR	10.1 ± 7.3	9.2 ± 5.67	10.1 ± 6.3^**^	0.175	0.023	0.668
	PMR	6.2 ± 2.6	7.4 ± 5.10	8.2 ± 7.07			
	DAL	11.1 ± 13.1	12.5 ± 10.0	13.1 ± 12.1^**^			
CK (U/L)	CMR	192.8 ± 132.1	237.6 ± 159.0	208.1 ± 144.6	0.492	0.403	0.117
	PMR	248.5 ± 122.9	304.0 ± 120.6	274 ± 111.8			
	DAL	208.2 ± 75.4	225.3 ± 52.9	210.8 ± 60.8			

Values expressed in Mean ± SD; Analyzed data according to GEE

*CMR* carbohydrate mouth rinsing, *PMR* placebo mouth rinsing, *DAL* drinking ad libitum

^a^time; ^b^ treatment; ^c^ interaction time * treatment; *Bonferroni* Post hoc: *p = 0.03; ^**^p = 0.01

## Discussion

The present study investigated the effect of CMR on biochemical and psychophysiological variables of cyclists in a fed state. There was no significant improvement of performance or change in metabolic response after CMR compared to the ad libitum strategy and the placebo. However, there was an attenuation on psychophysical responses after mouth rinse interventions (PMR and CMR) compared to ad libitum intake. Ad libitum water ingestion also led to higher blood lactate concentrations during the trial.

Recent studies indicate that the nutritional status prior to interventions is one of the factors determining improvement in cycling performance. Several studies report their benefit in 4 h fasting situations [12, 16–18, 29]. One possible explanation is that fasting can increase muscle metabolism in trained cyclists [32], and carbohydrates mouth rinse may enhance exercise intensity and promote more rapid muscular adaptations [33]. Nevertheless, the practice of competing while fasting is not common among athletes, and the relevance of this strategy is questionable [23]. In our study, the time trial was performed around 2 h after participants’ last meal, and there was no statistical change in performance, considering time for completion of test, heart rate, power and average speed. Similarly, researchers found no effect on performance when carbohydrate mouth rinse was used by athletes in a fed state [21–23]. It is important to note that athletes’ eating patterns did not vary during interventions on different days, which ensures that changes in the variables can be attributed to the strategies tested.

Regarding psychophysiological responses, no significant difference was observed between CMR and PMR, but both presented difference from ad libitum intake. RPE was higher in last 7.5 km and affective response was lower at 22.5 km in the DAL strategy. These results corroborates the higher lactate level found in the ad libitum intake. In contemporary theoretical models of fatigue, perceived exertion appears as an important indicator in the regulation of physical performance [34], which is used to monitor and to evaluate exercise tolerance and the level of effort, associating with the physiological stress [35]. The central governor theory proposes the existence of a conscious regulation in order to ensure the maintenance of the physiological homeostasis and prevent catastrophic changes. In this sense, previous study has found an association between increased blood lactate concentration and increased perceived exertion, suggesting a possible increment in muscle activity during increasing intensity of exercise [36]. Thus, a raise of perceived exertion may modulate performance by means of self-determined fluctuations in external workload, in order to prevent an unsustainable effort that would be deleterious to performance [34, 37, 38]. In our study is possible that participants under ad libitum intervention may have made a greater effort to maintain the same workload [12, 39], since no significant changes were found in power and performance of these individuals.

Previous studies did not find differences in perceptual responses between CMR and PMR interventions [12, 17, 23]. Backhouse et al. observed lower values of RPE when subjects ingested a carbohydrate solution compared to a placebo solution, but they did not report attenuation of perceptual responses when the solution is not ingested [40]. In our study, the use of artificial sweetener in order to reduce the difference of taste among solutions may have influenced the results, since the interaction of such sweeteners with taste receptors could mask the effect of carbohydrate solution [41, 42].

Cramer et al. believed that CMR would attenuate perceptual responses in the heat, however, they concluded that the high temperature may have counteracted any possible ergogenic effect of this strategy, since in these situations the high thermal and cardiovascular strain contribute more to performance than the presence of carbohydrates [43]. Studies previous have shown that the hydration status is not the main factor influencing physical performance and causing perceptual changes in this type of exercise [9, 10, 38, 44, 45]. In our study, subjects did not reach dehydration in any of the interventions, and fluid ingestion or rinsing did not influence the results. Goulet et al. suggested that exercise intensity and duration have a greater impact on performance [44].

In the pioneering study of Carter et al., suggested that CMR strategy could increase blood glucose and insulin concentrations, improving performance through enhanced glucose uptake into the active muscles and maintaining carbohydrate oxidation rates compared with water rinsing [12]. This hypothesis is based on the parasympathetic reflex and the cephalic phase of insulin release that are triggered by contact of sweet substances with taste receptors. However, the activation of theses mechanisms without the use of sweet substances has not been described [46]. Recently, Murray et al. [47] observed that CMR improved performance in 40 km cycling time-trial without altering plasma insulin concentration. Plasma insulin was collected at 5 km intervals throughout the first 25 km, and glucose samples were collected at 5 km intervals throughout the exercise bout. No change in plasma insulin was detected between conditions (*p* = 0.638, ES < 0.03). In our study, the carbohydrate used for mouth rinsing was a maltodextrin without flavor solution that was compared to rinsing with a placebo and ad libitum water intake, but no statistical differences were observed in glucose and insulin concentrations. Other studies also did not show significant changes in glucose concentrations without ingestion of carbohydrate solutions [17, 21, 29, 41].

Ali et al. compared strategies of mouth rinse and ingestion with carbohydrate and placebo, and they observed an increase in blood glucose, lactate and insulin concentrations only after carbohydrate intake [33]. Our study also suggested a lack of changes in metabolic responses under CMR, since we did not observe significant changes in plasma cortisol levels after exercise under the influence of all interventions. Bakhouse et al. reported a decrease in cortisol levels when carbohydrate was ingested, compared to placebo intake [40]. There are no other studies evaluating the effect of CMR on cortisol or other glucoregulatory hormones. Similarly, blood CK concentrations did not differ among interventions. These results support the hypothesis that the mechanism responsible for performance improvement observed in some studies is probably related to the central nervous system [2, 14, 18, 19]. Like most studies in the literature that evaluate the response of glucoregulatory hormones to exercise, we did not control plasma shifts accounted. In fact, we do not know whether these hormones are altered by possible plasma volume alterations induced exercise, so we suggest that future studies control this variable.

It is important to emphasize that the inclusion of the DAL strategy is a novelty of our study, comparing the ergogenic effects of CMR with the traditional fluid intake recommendations for this type of exercise [9–11]. Most studies compared CMR to placebo or water rinsing. Although we have not used scales of thirst sensation and gastrointestinal discomfort, participants reported that they preferred trials without rinsing, since they did not have the habit of using it. The sensation of thirst was expressed more after rinsing interventions, and there was no report of gastrointestinal discomfort in any of the interventions.

## Conclusions

CMR in a fed state showed no improvement in performance compared to placebo rinse and ad libitum water intake, with regard to time for completion of the test. RPE was higher and affective response was lower in only one moment of time trial of DAL intervention. CMR strategy did not influence the plasma levels of metabolic variables such as glucose, lactate, insulin, cortisol and CK. These findings suggest that the possible ergogenic effect of CMR does not exceed the effects of traditional fluid intake recommendations for cycling time trial.

## Abbreviations

ANOVA: Analysis of variance
BMI: Body mass index
CK: Creatine kinase
CMR: Carbohydrate mouth rinse
DAL: Drinking “ad libitum”
GEE: Generalized estimating eqs.
HR: Heart rate
PMR: Placebo mouth rinse
RPE: Rate of perceived exertion
VO_2máx_: maximum oxygen consumption
